# Anastral spindle assembly and γ-tubulin in *Drosophila *oocytes

**DOI:** 10.1186/1471-2121-12-1

**Published:** 2011-01-05

**Authors:** Sharyn A Endow, Mark A Hallen

**Affiliations:** 1 Department of Cell Biology and Structural Biology & Biophysics Program, Duke University

## Abstract

**Background:**

Anastral spindles assemble by a mechanism that involves microtubule nucleation and growth from chromatin. It is still uncertain whether γ-tubulin, a microtubule nucleator essential for mitotic spindle assembly and maintenance, plays a role. Not only is the requirement for γ-tubulin to form anastral *Drosophila *oocyte meiosis I spindles controversial, but its presence in oocyte meiosis I spindles has not been demonstrated and is uncertain.

**Results:**

We show, for the first time, using a bright GFP fusion protein and live imaging, that the *Drosophila *maternally-expressed γTub37C is present at low levels in oocyte meiosis I spindles. Despite this, we find that formation of bipolar meiosis I spindles does not require functional γTub37C, extending previous findings by others. Fluorescence photobleaching assays show rapid recovery of γTub37C in the meiosis I spindle, similar to the cytoplasm, indicating weak binding by γTub37C to spindles, and fits of a new, potentially more accurate model for fluorescence recovery yield kinetic parameters consistent with transient, diffusional binding.

**Conclusions:**

The FRAP results, together with its mutant effects late in meiosis I, indicate that γTub37C may perform a role subsequent to metaphase I, rather than nucleating microtubules for meiosis I spindle formation. Weak binding to the meiosis I spindle could stabilize pre-existing microtubules or position γ-tubulin for function during meiosis II spindle assembly, which follows rapidly upon oocyte activation and completion of the meiosis I division.

## Background

Anastral spindles assemble without centrosomes by a pathway that is still not fully understood. In particular, the mechanism by which microtubule nucleation occurs has not been well defined. Although chromatin has been shown to play an essential role [1], the involvement of the microtubule nucleator, γ-tubulin, is still an open question. γ-Tubulin localizes to centrosomes and other microtubule organizing centers in mitosis and is essential for nucleating microtubules in organisms as diverse as yeast, *Drosophila*, *Xenopus*, humans, and higher plants [2-5]. γ-Tubulin is also found on spindle microtubules, where it has been proposed to nucleate microtubules for spindle maintenance by functioning in a chromatin-mediated nucleation pathway that augments the dominant pathway of nucleation by centrosomes [6,7].

γ-Tubulin is present in cells as a large ring complex, γTuRC, comprising 12-13 γ-tubulin molecules associated with as many as ~7-8 other proteins [8,9]. γTuRC forms from a small complex, γTuSC, consisting of γ-tubulin bound to two other proteins [10-12]. The mechanism by which γTuRC nucleates microtubules is uncertain - it could serve as a ring-like template for a 12-or 13-protofilament microtubule [13,14] or, alternatively, α/β tubulin dimers could assemble onto an end of the large ring complex to form individual protofilaments [15].

Although γ-tubulin is believed to play a central role in mitotic spindle assembly and maintenance in many organisms, its role in anastral spindles that lack centrosomes is less certain. Analysis of mutants that affect the *Drosophila *oocyte- and early embryo-specific γTub37C [16] has led to the conclusion that γ-tubulin plays an essential role in nucleating microtubules for anastral oocyte meiosis I (MI) spindle assembly [17]. Conflictingly, another study of the same mutants concluded that γ-tubulin is not required for oocyte MI spindle formation [18]. Moreover, attempts by several groups to stain oocyte MI spindles using anti-γTub37C antibodies have produced negative results [17,19,20], raising doubts as to whether γ-tubulin is even present in the spindle.

Failure to localize γ-tubulin to MI spindles might be due to inadequate permeabilization of the dense cytoplasm of fixed *Drosophila *oocytes, hindering antibody staining, rather than the absence of γ-tubulin in spindles. A way around this problem is to analyze oocytes of flies expressing γ-tubulin tagged with an easily detectable fluorescent marker. Here we show using transgenic flies expressing γTub37C fused to a bright green fluorescent protein (GFP) that γ-tubulin is present at low levels in oocyte MI spindles. Nonetheless, bipolar MI spindles are present in *γ-tubulin *mutant oocytes, indicating that γ-tubulin is not required to form oocyte MI spindles. Fluorescence recovery after photobleaching (FRAP) assays demonstrate that γ-tubulin binding interactions with the spindle are weak and transient. γ-Tubulin is thus not required for MI spindle formation and binds weakly to the spindle; this may be important in stabilizing pre-existing microtubules or positioning γ-tubulin for function subsequent to MI spindle assembly.

## Results

### γTub37C localizes to oocyte MI spindles

Females expressing γTub37C fused to a bright GFP, regulated by the kinesin-14 *ncd *oocyte- and early embryo-specific promoter [21] were analyzed for this study. The females also expressed endogenous γTub37C without GFP, which is maternally loaded into the oocytes like Ncd. Western blot analysis showed lower levels of γTub37C-GFP than endogenous γTub37C in both adult females and ovaries (Additional file 1 Figure S1). Quantitation of the cross-reacting bands gave a ratio of 0.4-0.5 for γTub37C-GFP to γTub37C, indicating that the level of γTub37C-GFP in the transgenic flies is approximately half that of the endogenous γTub37C. This extends our previous estimate of the expression level of γTub37C-GFP in early embryos by measurement of GFP fluorescence, which we found to be similar to Ncd-GFP regulated by the same promoter [21]. Thus, γTub37C-GFP is not highly over-expressed, as frequently occurs when transgenes are driven by *gal4 *or other inducible promoters.

**Figure 1:**
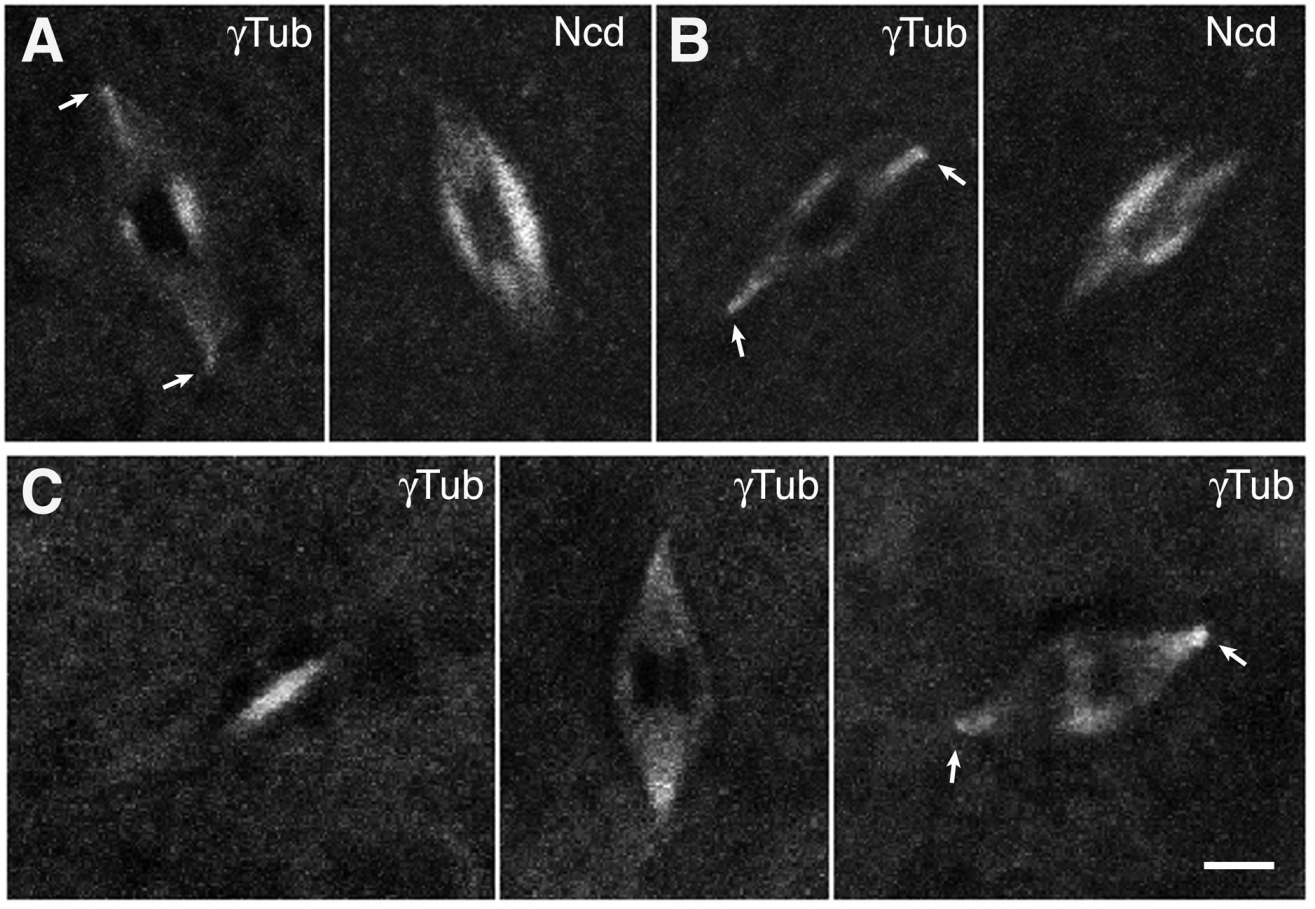
**γ-Tubulin localizes to oocyte MI spindles**. (A) MI spindle of a late stage 13 or (B) stage 14 oocyte expressing γTub37C-GFP (γTub, left) and Ncd-mRFP1 (Ncd, right). (C) MI spindles of a late stage 13 oocyte (left) or stage 14 oocytes (middle, right) expressing γTub37C-GFP but not Ncd-mRFP1. γ-Tubulin is present in the MI spindle midzone and at the spindle poles, including pole bodies (arrows). Bar, 3 μm.

Live oocytes were imaged to determine whether γ-tubulin is present in the MI spindle. Many, but not all of the oocytes showed detectable levels of γ-tubulin-GFP in late stage 13 (n = 16, total = 19) or stage 14 (n = 9, total = 11) spindles. γ-Tubulin localized differently to the spindles than Ncd-mRFP1, co-expressed by the females (n = 10) as a spindle marker [22]. It was typically found on the thick spindle equator, or spindle midzone, in late stage 13 oocytes and at the spindle poles, including the pole bodies, in stage 14 oocytes (Figure 1A). Its absence from spindles of some oocytes was attributed to the low levels of γ-tubulin in the spindle and the difficulty of detecting the protein. In addition, the endogenous unlabeled γTub37C in the oocytes probably occupies binding sites in the spindle, reducing the γTub37C-GFP fluorescence.

Oocytes of females (n = 7) expressing γTub37C-GFP, but not Ncd-mRFP1, were also examined to exclude the possibility of imaging artefacts caused by Ncd-mRFP1. γ-Tubulin was again found at low levels in late stage 13 (n = 5, total = 11) or stage 14 (n = 17, total = 18) MI spindles (Figure 1C), demonstrating detectable spindle localization even in the absence of another fluorescent protein in the spindle. The images of *γTub37C-gfp *oocyte spindles provide the first direct evidence that γ-tubulin is present in the *Drosophila *oocyte MI spindle. The low levels in the spindle and the difficulty in permeabilizing oocytes for antibody staining explain why previously reported antibody localization experiments did not produce positive results.

### γTub37C mutant embryos exhibit bipolar MI spindles

We then examined *γTub37C APL10 *mutant oocytes expressing Ncd-GFP as a spindle marker [23] to determine whether normal MI spindles were present. *γTub37C APL10 *mutant females lay eggs, but the eggs fail to hatch due to early embryo lethality [24]; the lethality is fully penetrant and can be used as indicator of *APL10 *homozygosity. We confirmed this by mating females in single pairs and monitoring vials for matings that produced embryos but no larvae, then extracting DNA from the female carcasses for PCR and γTub37C DNA sequence analysis after ovaries were removed for oocyte spindle imaging.

**Figure 2:**
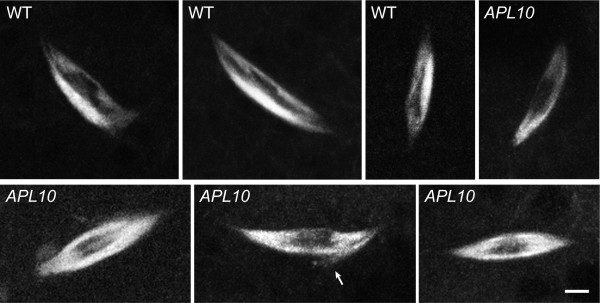
**MI spindles in *γTub37C APL10 *mutant oocytes**. *γTub37C APL10 *mutant oocytes contained bipolar MI spindles, many of which were normal, resembling those in wild-type (WT) oocytes, although some were frayed (arrow) or had split poles (not shown). Spindles visualized by Ncd-GFP. Bar, 3 μm.

Fifteen females produced embryos none of which hatched; bipolar spindles (n = 30, total = 32) were observed in oocytes from 10 females (Figure 2). Five females did not exhibit GFP-labeled spindles because the *ncd-gfp *transgene, which was on a chromosome that was segregating in the stock, was absent, as confirmed by PCR. Eleven of the 15 females were homozygous for *APL10 *by sequence analysis; two females gave poor sequence due to the small amount of DNA that was recovered and two females that showed no GFP spindle labeling were not sequenced. As a control, a female that produced embryos that hatched into larvae was sequenced and found to be *APL10*/+. Early embryo lethality of *γTub37C APL10 *is thus fully penetrant, confirming earlier reports [18,24]. Despite this, *APL10 *mutant oocytes exhibited bipolar MI spindles, many of which appeared normal (n = 14), although some spindles had split poles (n = 8) or were frayed (n = 6) (Figure 2, arrow), or both (n = 2). A few spindles were not bipolar (n = 2), resembling those of severe loss-of-function *ncd *mutants, which are defective in assembly [20].

Some of the abnormal MI spindles could have arisen because of insufficient Ncd, given that approximately a fourth of the fifteen *γTub37C APL10 *females were expected to be homozygous for the *ncd-gfp *transgene needed to image the spindles and the *ncd *null mutant *ca^nd^*, and two copies of the transgene do not completely rescue *ca^nd ^*[21-23,25]. However, all ten *γTub37C APL10 *females with GFP-labeled spindles appeared to carry a wild-type *ncd^+ ^*gene by PCR analysis, and thus should have been wild-type for *ncd *[20], producing infrequent Ncd-related spindle abnormalities (see below). Despite this, all ten females yielded one or more oocytes with an abnormal spindle.

Split poles or frayed spindles can also appear in wild-type oocytes - a previous study of wild-type *ncd-gfp *oocytes showed normal bipolar MI spindles (n = 15, total = 17) with a few spurred or multipolar spindles (n = 2) [26]. Some of these spindle abnormalities arise when imaging MI spindles in the final stages of assembly while the chromosomes are still undergoing movements [20] or due to inadvertent activation of oocytes while preparing them for imaging. However, the high frequency of abnormal *γTub37C APL10 *spindles (n = 18, total = 32) and the nature of the abnormalities (split poles, frayed spindles, defective assembly) raise the possibility that γ-tubulin may play a role in stabilizing MI spindles during assembly or ensuring correct spindle morphology.

Nonetheless, the presence of a significant proportion of normal-appearing bipolar MI spindles in *APL10 *mutant oocytes (~44%) indicates that γTub37C does not appear to be essential for the assembly of bipolar MI spindles. This conclusion was also reached in an earlier study [18], which reported that all the *γTub37C *mutants examined, including the null mutant, assembled bipolar oocyte spindles. However, this study, as well as other previous studies, did not demonstrate the presence of γ-tubulin in the MI spindle, as noted above.

### A mutant γTub37C does not bind to MI spindles

Several *γTub37C *mutants have been reported, including the *APL10 *allele [24] used in this study. These mutants cause female sterility due to early embryo lethality, providing strong evidence that the mutated residues are required for γTub37C function. The alleles that have been sequenced, including *APL10 *(E117K) [27], affect γ-tubulin residues that are conserved from fly to human. We tested the cytological effects of mutating a conserved γTub37C residue by recovering transgenic flies expressing an *APL10*-like mutation, *E116R-gfp*, immediately adjacent to E117, which is mutated in *APL10*. Both E116 and E117 are conserved in human γ-tubulin as E116 and D117, and are present in γTub37C helix H3, a helix that is thought to form lateral interactions between γ-tubulin molecules in γTuRC [28].

**Figure 3:**
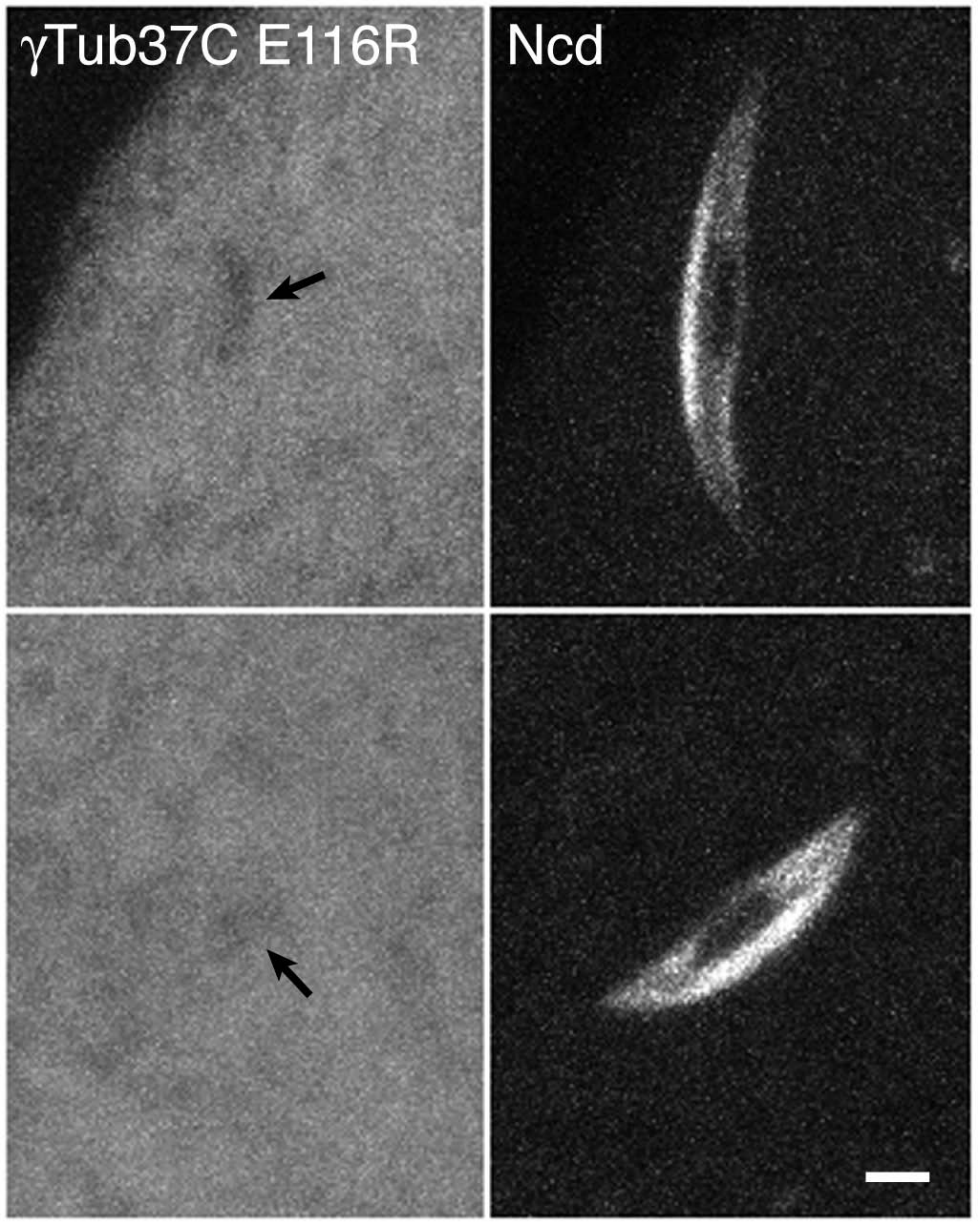
**Mutant γTub37C in oocytes**. γTub37C E116R-GFP, mutated in a conserved γTub37C residue, is present in the cytoplasm, but does not localize to oocyte MI spindles (left). Dark regions (arrows) correspond to the condensed MI chromosomes [23]. Spindles detected with Ncd-mRFP1 (right). Bar, 3 μm.

Live oocytes of *γTub37C*^+ ^females (n = 6) expressing γTub37C E116R-GFP together with Ncd-mRFP1 to visualize MI spindles showed GFP fluorescence dispersed throughout the cytoplasm, but no GFP localized to late stage 13 (n = 5) or stage 14 spindles (n = 11) (Figure 3). The dark region in the center of the spindle corresponds to the condensed chromosomes, which exclude fluorescence [23]. The dark region was detected in *γTub37C E116R-gfp *oocytes (Figure 3, arrows), but γTub37C E116R-GFP was not observed in spindles above cytoplasmic levels, in contrast to γTub37C-GFP, which was specifically localized to the MI spindle.

To estimate the expression level of the mutant relative to wild-type protein, γTub37C E116R-GFP cytoplasmic fluorescence was normalized to the dark chromosomal region and compared to γTub37C-GFP cytoplasmic fluorescence normalized in the same way. This analysis showed comparable cytoplasmic fluorescence for γTub37C E116R-GFP (1.51 ± 0.03 a.u., n = 6) and γTub37C-GFP (1.31 ± 0.04 a.u., n = 6). Western blot analysis also showed similar levels of γTub37C E116R-GFP and γTub37C-GFP compared to γTub37C in ovaries (Additional file 1 Figure S1). Thus, the mutant protein is expressed and is present in the cytoplasm at levels similar to the wild-type protein; despite this, it does not localize to MI spindles. The ability to detect γTub37C-GFP but not γTub37C E116R-GFP in oocyte MI spindles indicates that γTub37C binds specifically although at low levels to the spindle midzone and poles, rather than being present in spindles simply because it diffuses into the spindle from the cytoplasm. It also shows that mutating a conserved residue perturbs the localization of γTub37C to the spindle, possibly due to the failure to form higher-order γ-tubulin complexes.

Although the *γTub37C APL10 *mutation, E117K, differs from the *γTub37C *E116R mutant that we tested cytologically, it is similar in that it also affects a conserved, negatively charged residue in helix H3 and changes it to a positively charged residue. This means that *APL10 *could have a similar effect to γTub37C E116R in potentially disrupting γ-TuRC formation or other γTub37C interactions. Thus, despite the likelihood that the *APL10*-mutated γTub37C is not associated with the MI spindle, bipolar spindles can form, as observed in *APL10 *mutant oocytes. These observations indicate that assembly of bipolar MI spindles does not require functional γTub37C.

### γTub37C mutant embryos show effects subsequent to metaphase I

*γTub37C APL10 *mutant embryos were examined to determine the earliest stage at which *γ*Tub37C is required for function. Embryos, collected for ≥1.5-2 hr, were fixed and stained with rhodamine-α-tubulin antibody and DAPI to image the spindles and chromosomes, respectively. Ncd-GFP, co-expressed by the mutant females, was also used to visualize the spindles; in some cases, spindles were imaged only with Ncd-GFP. Ore R embryos, collected for 0-15 min, were fixed and stained for controls.

**Figure 4:**
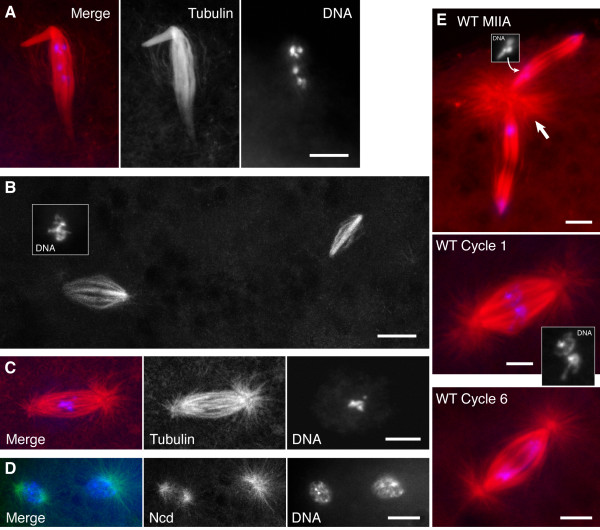
***γTub37C APL10 *mutant embryos**. (A) Anaphase I arrest. (B) Anastral spindles associated with MII metaphase chromosomes (inset) from an embryo containing 6 or more anastral spindles and a polar body with 1N chromosomes. (C) Astral cycle 1 mitotic spindle containing a haploid set of chromosomes. (D) Two small astral spindles associated with interphase or prometaphase nuclei from an embryo containing ~20 astral spindles (~cycle 6) and two pairs of MII-like anastral spindles without central spindle bodies. (E) Wild-type spindles. Top, anaphase II spindle with central spindle pole body (large arrow) and segregating haploid chromosome sets (inset); middle, cycle 1 mitotic spindle containing two haploid chromosome sets (inset); bottom, cycle 6 mitotic spindle. Tubulin, red; Ncd-GFP, green; DNA, blue. Bars, 5 μm.

Mutant embryos displayed complex abnormal spindle and chromosome configurations (Figure 4). Many embryos were blocked in stages that resembled late MI (n = 4, total = 13) or MII (n = 5). The earliest stage of arrest observed was anaphase I (Figure 4A). The spindles and chromosomes in a given embryo frequently differed from one another in stage, e.g., a polar body in one embryo was associated with condensed MI-like chromosomes. Multiple anastral spindles containing metaphase II-like chromosomes were present in others (Figure 4B), instead of the typical MII spindle of wild type, which comprises two tandem spindles joined by a central spindle pole body (Figure 4E, top).

Polar bodies in several embryos contained >2N chromosomes, indicating failure to undergo the meiotic divisions or continued chromosome divisions after MII. One embryo displayed a single large astral spindle associated with a haploid set of chromosomes (Figure 4C), which we interpreted to be a cycle 1 mitotic spindle from an embryo that had failed to undergo pronuclear fusion, by comparison with wild type (Figure 4E, middle). Another embryo exhibited many small astral spindles (Figure 4D) that were a third to half the size of wild-type spindles at approximately the same cleavage cycle (Figure 4E, bottom). The centrosomes associated with these spindles were presumably from the fertilizing sperm, but the small size of the spindles was consistent with their containing haploid, rather than diploid chromosome complements [29]. Spindles and chromosomes in many embryos had divided out of synchrony with each other, resulting in abnormal configurations that were difficult to interpret with certainty.

Overall, the earliest stage of arrest appeared to be anaphase I. Normal MII spindles were not observed and probably failed to form. Polar bodies could form even when the chromosomes had not completed the meiotic divisions. In some embryos, divisions occurred on multiple anastral spindles; in another, presumed haploid chromosome complements segregated on astral spindles. These observations are consistent with the interpretation that the earliest *γTub37C APL10 *mutant defects occur in late MI and normal MII divisions fail to occur, and that pronuclear migration and fusion may also be defective. These findings extend the conclusion reached by others in a previous study that *γTub37C *null mutant embryos may be arrested in meiosis [18].

### γ**Tub37C binds weakly to MI spindles**

**Figure 5:**
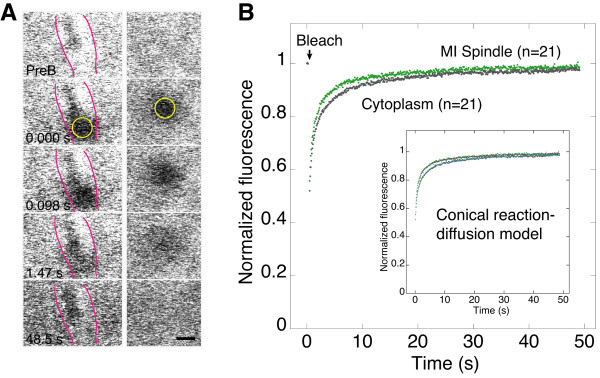
**Fluorescence photobleaching analysis of γTub37C in MI spindles**. (A) Prebleach (PreB), bleach and recovery images from FRAP assays of *γ*Tub37C-GFP in the oocyte MI spindle (left) and cytoplasm (right). Time, s. ROI (yellow circles; radius, w = 1.062 μm). The MI spindle (left) is outlined to show its boundaries (pink lines). Bar, 2 μm. (B) Mean normalized fluorescence over time (n = 21), corrected for photobleaching during the assays. For comparison, only the data for the large ROI are shown (spindle, green; cytoplasm, grey). Inset, fits to new reaction-diffusion FRAP recovery model that assumes a conical bleach profile (spindle, purple; cytoplasm, cyan).

γ-Tubulin binding interactions with oocyte MI spindles were analyzed by performing FRAP assays and fitting models for fluorescence recovery to the data to obtain values for binding and dissociation rate constants and the diffusion coefficient. A large or small region of interest (ROI) was bleached in a *γTub37C-gfp *oocyte MI spindle or the adjacent cytoplasm, and fluorescence recovery was monitored in the bleach spot [30]. The data were corrected for photobleaching during recovery imaging by adding back the fluorescence that was lost in an unbleached region of the spindle or cytoplasm. The recovery curves for the spindle and cytoplasm were similar to one another with slightly faster recovery for the spindle than cytoplasm (Figure 5). The small ROI in both the spindle and cytoplasm showed faster recovery than the large ROI, indicating that diffusion plays a dominant role in recovery.

The corrected data were fit to FRAP models to estimate kinetic parameters for γ-tubulin binding to the oocyte spindle or cytoplasm. The models tested were a diffusion phase-binding phase (previously referred to as diffusion-binding) and two-state binding dominant model [31], and a new conical reaction-diffusion model [32]. The diffusion phase-binding phase and two-state binding dominant models assume a cylindrical bleach profile, which is a reasonable approximation when recovery shows a binding-dominant phase that can be used to estimate kinetic parameters for binding interactions with cellular structures [21]. The new conical reaction-diffusion model assumes a double-cone bleach profile that more closely resembles the actual point spread function of a high numerical aperture objective like the one used in this study. The model represents an exact analytical solution using Fourier and Hankel transforms for fluorescence recovery in the bleach region that is expected to yield more accurate estimates of binding and diffusion parameters than previous models.

Visually good fits for all three models were obtained for the MI spindle and cytoplasm FRAP data by concurrently fitting the data for the large and small ROIs [21]. A comparison of the kinetic parameters from the models is given in Additional file 1 Table S1. Values from the new conical reaction-diffusion model for the oocyte spindle and cytoplasm data are also given below and in Table 1; we believe that they are likely to be the most accurate of the three models because the model assumes a more accurate photobleaching profile and because of the dominant role of diffusion in the recovery.

**Table 1:** γTub37C kinetic parameters in the spindle and cytoplasm

Spindle or cytoplasm	D(μm^2^/s)	k*_on _(s^-1^)	k_off _(s^-1^)	t^1^/_2 _(s)	C_eq_
**MI spindle**	12 ± 5	0.02 ± 0.02	0.6 ± 0.3	1.1	0.03 ± 0.03
**Ooplasm**	8 ± 3	0.14 ± 0.03	0.9 ± 0.1	0.8	0.14 ± 0.03
					
**Mitotic spindle**	28 ± 10	0.015 ± 0.006	0.23 ± 0.04	3.0	0.06 ± 0.02
**Embryo cytoplasm**	26 ± 8	0.009 ± 0.004	0.15 ± 0.03	4.5	0.05 ± 0.02

Kinetic parameters from fits to the FRAP data of the conical reaction-diffusion model [32]; the half-time of recovery was calculated by t^1^/_2 _= ln2/k_off _and the bound protein fraction at equilibrium by C_eq _= k*_on_/(k*_on_+k_off_) [31].

For γTub37C in the MI spindle, the diffusion coefficient, D = 12 ± 5 μm^2^/s; pseudo first-order binding constant, k*_on _= 0.02 ± 0.02 s^-1^; and dissociation constant, k_off _= 0.6 ± 0.3 s^-1^, giving the recovery half-time, t^1^/_2 _= 1.1 s, and bound protein fraction at equilibrium, C_eq _= 0.03 ± 0.03 (Table 1). In the oocyte cytoplasm or ooplasm, D = 8 ± 3 μm^2^/s, k*on = 0.14 ± 0.03 s^-1 ^and k_off _= 0.9 ± 0.1 s^-1^, giving t^1^/_2 _= 0.8 s and C_eq _= 0.14 ± 0.03. Thus, the bound state is somewhat more prevalent in the ooplasm than in the meiotic spindle, although the majority of protein is still freely diffusing. Binding to the MI spindle is very low. The diffusion coefficients for the MI spindle and ooplasm are not significantly different; the lower diffusion coefficients than for γTub37C in mitotic spindles and cytoplasm (see below and Table 1) reflect the more viscous ooplasm.

For comparison with the MI spindle results, we also fit the new conical reaction-diffusion model to our previous FRAP data for γTub37C in early embryo mitotic spindles [21] (Table 1). The parameters differed from those we obtained previously by fitting the data to a two-dimensional recovery model, but were of the same order of magnitude and were in agreement with our previous conclusions. In the mitotic spindle, D = 28 ± 10 μm^2^/s while k*_on _= 0.015 ± 0.006 s^-1 ^and k_off _= 0.23 ± 0.04 s^-1^, giving t^1^/_2 _= 3.0 s and C_eq _= 0.06 ± 0.02. In the embryo cytoplasm, D = 26 ± 8 μm^2^/s while k*_on _= 0.009 ± 0.004 s^-1 ^and k_off _= 0.15 ± 0.03 s^-1^, giving t^1^/_2 _= 4.5 s and C_eq _= 0.05 ± 0.02. Thus, binding was relatively weak in both the mitotic spindle and embryo cytoplasm, with a similar turnover time and the same small amount of protein bound. The diffusion coefficient did not differ significantly between the mitotic spindle and embryo cytoplasm, as we observed for the MI spindle and ooplasm.

The kinetic parameters from the FRAP data indicate that γTub37C binding to the MI spindle is transient with weak and diffusional properties, as in the ooplasm, similar to early embryo mitotic spindles and cytoplasm. This differs from the tight binding by γTub37C to centrosomes of mitotic spindles [21], where it functions to nucleate microtubules for spindle formation. The diffusion coefficient for γTub37C-GFP in the oocyte spindle of D = 12 ± 5 μm^2^/s is probably too high to be γTuRC, which we estimated to have a D value of 3-8 μm^2^/s in the embryo cytoplasm [21]; the D value in the oocyte spindle is expected to be even lower than this estimate because of the high viscosity of the ooplasm.

## Discussion

Microtubule nucleation for anastral spindle assembly is dependent on chromatin, differing from astral spindles; however, the mechanism of nucleation is still not certain. The initial stages in formation of anastral *Drosophila *oocyte MI spindles have been studied by imaging live oocytes injected with rhodamine-tubulin [33] or expressing the kinesin-14 Ncd motor fused to GFP to visualize the spindle [20]. Microtubule growth from the condensed MI chromosomes or karyosome was observed in both cases, providing evidence that microtubule nucleation occurs from chromatin, as first observed in *Xenopus *extract spindles [1]. Time-lapse images obtained with Ncd-GFP showed small fluorescent foci or asters that migrated towards and associated with the karyosome, after which spindle microtubules grew out in all directions from the chromosomes [20]. The asters were also observed labeled with rhodamine-tubulin but were thought not to play a role in spindle assembly, in part because they were not associated with spindle poles [33]. Their labeling by both Ncd-GFP and rhodamine-tubulin and behavior at early times following nuclear envelope breakdown are consistent with the interpretation that they consist of Ncd bound to small microtubules that nucleate microtubule growth after association with the chromosomes. Similar foci have been observed in early stages of spindle assembly in mouse oocytes [34].

Although the Ncd asters were interpreted as providing the nucleating activity for microtubule growth and MI spindle formation [20], it has still been a possibility that γ-tubulin, the microtubule nucleator for mitotic spindle assembly, plays a role. Two highly divergent γ-tubulin isoforms, γTub23C and γTub37C, exist in *Drosophila*. The two isoforms show 83% amino acid identity and are differentially expressed: γTub23C is found in both males and females, but is not present in nurse cells or oocytes and is observed in early embryos only after cellularization, whereas γTub37C is expressed only in females and accumulates in oocytes and early syncytially dividing embryos [16]. γTub37C, the *Drosophila *oocyte- and early embryo-specific γ-tubulin, has not previously been localized to oocyte MI spindles, but we show here that it is present at low levels in the spindle midzone and on the poles and pole bodies, differing from the kinesin-14 motor, Ncd, which is present throughout the spindle. Its distribution indicates that γ-tubulin binds specifically to the MI spindle, rather than simply diffusing into the spindle from the ooplasm, which is expected to result in failure to accumulate in the spindle, as observed for a γTub37C E116R mutant protein.

Despite its localization to the spindle, γ-tubulin shows transient binding interactions with the MI spindle by FRAP analysis. Fits of a new fluorescence recovery model, which is expected to yield more accurate values for binding parameters than previous models, gave a 30-fold higher k_off _than k*_on _for γTub37C binding to the MI spindle, indicating low binding affinity characterized by weak interactions. By comparison, the k_off _was ~6-fold greater than the k*_on _for γTub37C in the ooplasm, again indicating weak binding interactions but somewhat higher binding affinity than in the spindle. The diffusion coefficient, D, for γTub37C in the spindle did not differ significantly from the ooplasm.

The transient, weak binding by γTub37C to the MI spindle differs from its tight binding to centrosomes of mitotic spindles in early embryos [21], but is similar to the weak binding by γTub37C to the mitotic spindle and cytoplasm of early embryos, which show a 15-17 fold higher k_off _than k*_on_. D for γTub37C in the mitotic spindle and cytoplasm was the same, as for the MI spindle and ooplasm.

The weak binding by γTub37C to MI spindles implies that it is not nucleating microtubules, given that it dissociates with a turnover rate that is too fast for substantial assembly to have occurred. Alternatively, it could nucleate microtubule growth by a mechanism that differs from that of centrosomes. However, the finding of bipolar MI spindles in *γTub37C APL10 *mutant oocytes supports the interpretation that γTub37C is not required for MI spindle formation. Instead, microtubules for MI spindle formation are most likely nucleated by the Ncd-bound asters observed to migrate to and associate with the karyosome [20].

γTub37C is also present in oocyte MII spindles, where it is easily detectable by antibody staining, brightly labeling the central spindle pole body, which is thought to organize microtubules for rapid assembly of the two tandem MII spindles following anaphase I [35-37]. γ-Tubulin localization to the MII spindle is cell-cycle dependent - it is present in metaphase II but absent by late telophase II [35]. Although little is known about the recruitment of γ-tubulin to the MII spindle, the findings we report here suggest that weak binding by γ-tubulin to the MI spindle and its redistribution following anaphase I could account for its rapid appearance in the MII spindle following anaphase I. The MII spindle forms by reorganization of the anaphase I spindle without its disassembly - the central spindle pole body and new poles of the two tandem spindles form in the center of the anaphase I spindle [23]. Weak binding by γ-tubulin to the MI spindle midzone and poles may reflect the presence of interacting proteins that are positioned for function subsequent to anaphase I; their activation by phosphorylation or other modifications could account for the rapid sequence of events during progression of the oocyte MI and MII divisions.

Although the details of these events are likely to differ from organism to organism, basic features may be common to many organisms. For example, the finding of fluorescent asters in mouse oocytes that resemble those in *Drosophila *oocytes implies that the asters may be a general feature of "anastral" spindle formation. They were not reported in studies of *Xenopus *extract spindles, most likely because of the disruption of the cytoplasm during extract preparation. The asters are inferred from studies in *Drosophila *to consist of the kinesin-14 motor Ncd bound to short microtubules [17,19,20]; further studies are needed to characterize the asters and define their migration mechanism and role in microtubule nucleation.

## Conclusions

γ-Tubulin does not appear to be essential for MI spindle assembly but binds weakly to the MI spindle, potentially stabilizing pre-existing microtubules or interacting with other proteins positioned for function later in the meiotic divisions. Available evidence suggests that Ncd motor-associated microtubule asters observed in previous studies are the primary source of new microtubules in the MI spindle. Further study of these asters should provide information regarding microtubule nucleation during "anastral" spindle assembly.

## Methods

### Fly lines

Transgenic flies expressing γTub37C-GFP, γTub37C E116R-GFP, Ncd-GFP and Ncd-mRFP1 have been reported previously [21-23,25]. One or two copies of *γTub37C-gfp *fully rescues the female sterility of heterozygous *γTub37C APL10/DF(2L)VA23 *females which carry the *γTub37C APL10 *mutant allele over a deficiency that covers *γTub37C *[21]. Thus, *γTub37C-gfp *can complement a γ*Tub37C *mutant and appears to be functional. γTub37C-GFP and γTub37C E116R-GFP expression levels were estimated by Western blot analysis performed using conventional methods and total protein from *γTub37C-gfp *females or ovaries cross-reacted with anti-γTub37C antibodies (a gift of Y. Zheng); *w^1118 ^*host females and ovaries were used as controls. Cross-reacting bands were quantitated using NIH ImageJ.

### Live imaging

*γTub37C APL10 *mutant females were mated in single pairs and vials were monitored for embryo hatching up to 2-3 weeks. Ovaries were removed after several days of egg laying and remains of the females were quick frozen for DNA analysis. Live late stage 13 and stage 14 oocytes were prepared from the ovaries as described previously [20,30]; spindles were imaged by laser-scanning confocal microscopy using a Bio-Rad Radiance2100 confocal scanhead mounted on a Zeiss Axioskop 2 Plus microscope, LaserSharp 2000 software, a 40x/1.3 NA Plan-NeoFluar oil immersion objective, and the 488-nm line of a 10 mW Kr-Ar laser. Co-expressed Ncd-GFP was used to visualize the spindles, which were analyzed for bipolarity and overall structure by comparison to spindles from control wild-type *ncd-gfp *oocytes. Females that produced embryos that failed to hatch were confirmed homozygous for *APL10 *by PCR and DNA sequence analysis.

### Fixed embryos

*γTub37C APL10 *females were mated in single pairs to obtain embryos for analysis of mutant effects; vials were monitored for absence of embryo hatching to confirm the females were homozygous for *APL10*. Embryos were collected 1^1^/_2_-2 hr; a few embryos that were analyzed were collected over longer time periods, either 4 hr or overnight. Embryos were dechorionated manually in batches and incubated ~2 min in heptane, washed in PBS and fixed for ~3 min in 4-8% formaldehyde in PBS, then washed and stored in PBS at 4°C. Vitelline membranes were removed manually prior to staining with rhodamine-labeled α-tubulin antibody and/or DAPI. Spindles were imaged by rhodamine and/or co-expressed Ncd-GFP fluorescence using the Bio-Rad Radiance2100 confocal microscope described above; chromosome images were acquired using a Hamamatsu Orca-*R^2 ^*cooled CCD camera attached to a Leitz Dialux 22 fluorescence microscope using a 63x/1.40 NA PL APO oil immersion objective. For controls, Ore R embryos collected 0-15 min were fixed and stained with rhodamine-labeled α-tubulin antibody and DAPI.

### FRAP assays

FRAP assays of *γTub37C-gfp F13F3 *oocyte MI spindles and cytoplasm were performed at ~22°C using a Zeiss LSM 510 confocal microscope and LSM 510 software, a 40x/1.3 NA Plan-NeoFluar oil immersion objective and the 488-nm line of a 30 mW Ar laser set at 50% power, as described [30]. Assays, performed at zoom 3, consisted of three prebleach images, four rapid bleach scans at full laser power (ROI diameter = 29 or 14 pixels; radius, w = 1.062 or 0.513 μm at 13.653 pixels/μm) and 497 recovery images at 98 ms/image and low laser power (1.5%). Fluorescence intensity in the large or small bleach spot over time recorded by the Zeiss 510 software was averaged for repeated assays (n = 21) of the same or different spindle or cytoplasm (3 oocytes from 3 females), except that the small ROI for the spindle consisted of fewer assays (n = 10) of the spindle in 1 oocyte. Fluorescence loss during the assay due to photobleaching was measured in unbleached regions of the spindle or cytoplasm, averaged and added back to the mean recovery data to correct for photobleaching. After correction, the mean data for the large and small ROIs were fit to FRAP recovery models to estimate binding and dissociation rate constants for γTub37C in the MI spindle and cytoplasm.

FRAP data for the spindle and cytoplasm in oocytes, acquired for the present study, and in early embryos, taken from a previous study [21], each with two different bleach spot sizes, were fit to fluorescence recovery models to determine binding and diffusion parameters. One of these was a new FRAP model that assumes a conical bleach profile approximating the point spread function of a high NA objective in a confocal microscope [32]. It has five parameters for each recovery curve: the diffusion coefficient D, pseudo-first-order association rate constant k*_on_, dissociation rate constant k_off_, bleach constant k_b _(which indicates the extent of bleaching), and total recovery R (which indicates the amount of fluorescence when recovery is complete and thus provides a normalization for the curve). This number of parameters is too many to extract accurately from a single recovery curve; a concurrent fitting strategy [21], in which parameters are shared between recovery curves, was thus used to allow accurate fitting.

The curve fitting consisted of two fits, one for the oocyte data and one for the early embryo data, since the assay conditions differed somewhat for the data sets and no parameters were assumed shared between the data sets. However, the large and small bleach spots in the spindle or cytoplasm were assumed to have the same values of D, k*_on_, and k_off_, since these are properties of γTub37C in each cellular compartment and are not related to the bleaching. Furthermore, the large bleach spots in the spindle and cytoplasm were considered to share a value of k_b_, as were the small bleach spots, although these values are different in the oocyte and early embryo data sets because the bleaching conditions differed somewhat. Each curve had its own value of R. Thus, each fit extracted twelve parameters from four data sets; a 3:1 parameter-to-data-set ratio is reasonable for nonlinear data like a FRAP recovery. The *nlinfit *function in MATLAB was used for the fitting, which was performed on the Duke University Blue Devil Grid. Because a greater amount of statistical error occurred in some data sets than others (for example, in smaller bleach spots), the robust option in *nlinfit *was used; this assigns greater weight to data with less error.

## Abbreviations

FRAP: fluorescence recovery after photobleaching; GFP: green fluorescent protein; MI: meiosis I; MII: meiosis II; ROI: region of interest.

## Authors' contributions

SAE designed and performed experiments, analyzed results and wrote the manuscript draft; MAH fit the FRAP data to models, interpreted and wrote the results, and helped revise the manuscript. Both authors read and approved the final manuscript.

## Supplementary Material

Additional file 1 **γTub37C-GFP expression levels in transgenic flies and comparison of kinetic parameters from different FRAP models fit to the spindle and ooplasm data**. Figure S1 shows a Western blot of wild-type and mutant γTub37C-GFP expression levels in transgenic flies and ovaries compared to endogenous γTub37C; Table S1 shows a comparison of γTub37C kinetic parameters in the MI spindle and ooplasm derived from fits of different fluorescence recovery models to the FRAP data. Click here for file
