# Supplementary material for: Anastral spindle assembly and γ-tubulin in Drosophila oocytes
Source: BMC Cell Biol. 2011 Jan 5;12:1. doi: 10.1186/1471-2121-12-1 (PMC3022845; doi:10.1186/1471-2121-12-1)
Supplement: Additional file 1 — γTub37C-GFP expression levels in transgenic flies and comparison of kinetic parameters from different FRAP models fit to the spindle and ooplasm data. Figure S1 shows a Western blot of wild-type and mutant γTub37C-GFP expression levels in transgenic flies and ovaries compared to endogenous γTub37C; Table S1 shows a comparison of γTub37C kinetic parameters in the MI spindle and ooplasm derived from fits of different fluorescence recovery models to the FRAP data. [file 1471-2121-12-1-S1.PDF]

Anastral spindle assembly and γ-tubulin in *Drosophila* oocytes

Sharyn A. Endow and Mark A. Hallen

Department of Cell Biology and Structural Biology & Biophysics Program, Duke University Medical Center, Durham, NC 27710

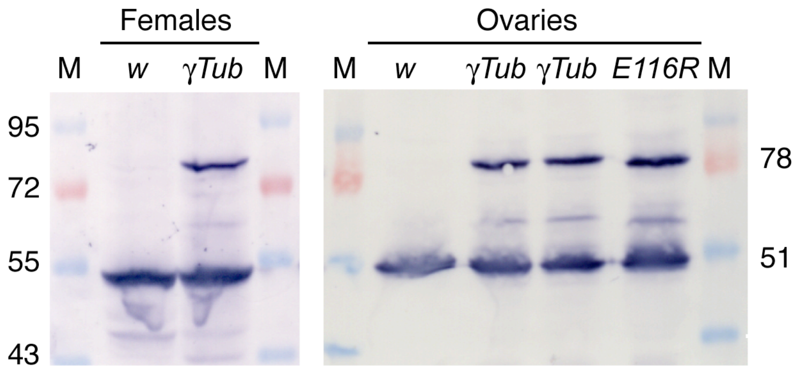

Figure S1 γTub37C-GFP expression levels in transgenic flies. Western blot of total protein from *w*[1118] (*w*; host fly) or *γTub37C-gfp* (*γTub*) adult females (3), or *w*[1118], *γTub37C-gfp* or *γTub37C E116R-gfp* (*E116R*) ovaries (3 pairs) cross-reacted with anti-γTub37C antibodies. Major bands are γTub37C-GFP (78 kDa) and γTub37C (51 kDa). Ratios of γTub37C-GFP to γTub37C are 0.4-0.5 by quantitation with NIH ImageJ. M, molecular mass markers (kDa).

Table S1: Comparison of kinetic parameters from different FRAP models fit to the spindle and ooplasm data

Diffusion-binding models

Parameter	Conical model	Cylindrical model
**Spindle D ($\mu m^2/s$)**	12±5	1.29±0.06
**Spindle k^*_{on} (s^{-1})**	0.02±0.02	0.0009±0.0009
**Spindle k_{off} (s^{-1})**	0.6±0.3	0.03±0.02
**Spindle C_{eq}**	0.03±0.03	0.03±0.01
**Ooplasm D ($\mu m^2/s$)**	8±3	1.58±0.09
**Ooplasm k^*_{on} (s^{-1})**	0.14±0.03	0.021±0.002
**Ooplasm k_{off} (s^{-1})**	0.9±0.1	0.16±0.01
**Ooplasm C_{eq}**	0.14±0.03	0.119±0.007

Two-state binding model

Parameter	Slow binding phase	Fast binding phase
**Spindle k^*_{on} (s^{-1})**	0.033±0.003	1.2±0.1
**Spindle k_{off} (s^{-1})**	0.102±0.006	0.98±0.05
**Spindle C_{eq}**	0.129±0.006	0.48±0.02
**Ooplasm k^*_{on} (s^{-1})**	0.056±0.004	1.3±0.1
**Ooplasm k_{off} (s^{-1})**	0.109±0.005	1.04±0.05
**Ooplasm C_{eq}**	0.182±0.006	0.46±0.02

The major difference between the models is the presence of the fast binding phase in the two-state binding model; the protein in this phase is treated more or less as free protein by the other models, which have only the slower binding phase. For this phase, C_{eq} is most similar between the models; the two diffusion-binding models agree on the C_{eq} values, even though the rate constants differ by 6-20 fold. Note that C_{eq} in the two-state binding model is derived from the curve fit, rather than calculated from k^*_{on} and k_{off}, as in the other two models. The diffusion coefficients estimated by the cylindrical and conical models differ; however, the cylindrical D values are both 5-10 times smaller than the conical values. The error bounds are wider for the conical model than the others; these bounds are likely to be more realistic. They reflect the greater number of parameters (allowing a more accurate description of the system) and greater extent of concurrent fitting possible with these parameters.
